# A chloride ring is an ancient evolutionary innovation mediating the assembly of the collagen IV scaffold of basement membranes

**DOI:** 10.1074/jbc.RA119.007426

**Published:** 2019-03-28

**Authors:** Vadim Pedchenko, Ryan Bauer, Elena N. Pokidysheva, Alaa Al-Shaer, Nancy R. Forde, Aaron L. Fidler, Billy G. Hudson, Sergei P. Boudko

**Affiliations:** From the a Department of Medicine, Division of Nephrology and Hypertension,; b Vanderbilt Center for Matrix Biology, Vanderbilt University Medical Center, Nashville, Tennessee 37232,; the Departments of c Molecular Biology and Biochemistry and; d Physics, Simon Fraser University, Burnaby, British Columbia V5A 1S6, Canada,; the e Aspirnaut^TM^ Program,; Departments of f Pathology, Microbiology, and Immunology and; g Cell and Developmental Biology, Vanderbilt University Medical Center, Nashville, Tennessee 37232, and; the h Department of Biochemistry,; i Vanderbilt-Ingram Cancer Center,; j Vanderbilt Institute of Chemical Biology, Vanderbilt University, Nashville, Tennessee 37232

**Keywords:** collagen, basement membrane, protein self-assembly, crystal structure, extracellular matrix, kinetics, connective tissue, animal evolution, chloride, collagen IV, ECM, NC1 domain

## Abstract

Collagen IV scaffold is a principal component of the basement membrane (BM), a specialized extracellular matrix that is essential for animal multicellularity and tissue evolution. Scaffold assembly begins with the trimerization of α-chains into protomers inside the cell, which then are secreted and undergo oligomerization outside the cell. For the ubiquitous scaffold composed of α1- and α2-chains, both intracellular and extracellular stages are mediated by the noncollagenous domain (NC1). The association of protomers is chloride-dependent, whereby chloride ions induce interactions of the protomers' trimeric NC1 domains leading to NC1 hexamer formation. Here, we investigated the mechanisms, kinetics, and functionality of the chloride ion-mediated protomer assembly by using a single-chain technology to produce a stable NC1 trimer comprising α1, α2, and α1 NC1 monomers. We observed that in the presence of chloride, the single-chain NC1-trimer self-assembles into a hexamer, for which the crystal structure was determined. We discovered that a chloride ring, comprising 12 ions, induces the assembly of and stabilizes the NC1 hexamer. Furthermore, we found that the chloride ring is evolutionarily conserved across all animals, first appearing in cnidarians. These findings reveal a fundamental role for the chloride ring in the assembly of collagen IV scaffolds of BMs, a critical event enabling tissue evolution and development. Moreover, the single-chain technology is foundational for generating trimeric NC1 domains of other α-chain compositions to investigate the α121, α345, and α565 collagen IV scaffolds and to develop therapies for managing Alport syndrome, Goodpasture's disease, and cancerous tumor growth.

## Introduction

The fundamental architectural unit of metazoan epithelial tissues is characterized by a layer of apical/basal-polarized cells that are laterally connected by tight junctions between plasma membranes and basally anchored via integrin receptors to a basement membrane (BM)^3^ (1, 2). The underlying BM, a specialized form of extracellular matrix (ECM), provides structural integrity to tissues, guides cell migration and adhesion, delineates apical–basal polarity, and modulates cell differentiation during development (3–5). BM was a key innovation enabling animal multicellularity (1, 6). How BMs function and how they are assembled on the outside of cells remain paramount knowledge gaps in cell biology (7).

**Figure 1.**
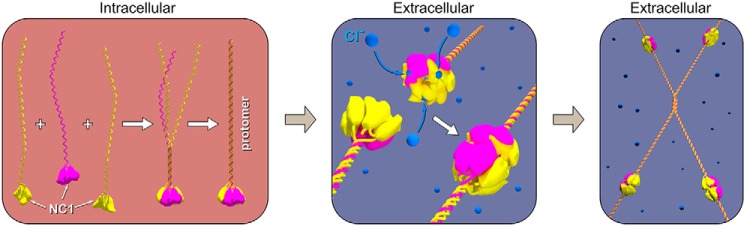
**Assembly of collagen IV scaffolds.** Protomer assembly occurs within the cell, where two α1-chains (*yellow*) and one α2-chain (*violet*) associate into a trimer via the globular NC1 domain followed by triple-helix formation in a zipper-like mode. Once secreted outside the cells, chloride concentration triggers collagen IV protomers to oligomerize end–to–end via NC1 trimer–to–trimer association forming an NC1 hexamer bridging two protomers. Scaffold assembly also involves the end–to–end oligomerization of four protomers forming a tetramer connected by a 7S domain.

BMs are assembled from a toolkit of proteins that includes a collagen IV scaffold as a principal component (1, 6, 8, 10). This scaffold confers structural integrity to tissues, binds integrins for cell adhesion and signaling, binds bone morphogenic proteins for signaling gradients during tissue development, and tethers a diverse assortment of molecules, including laminins, proteoglycans, and growth factors, which harbor a plethora of functions (3, 4, 11–15). Scaffold assembly involves two stages of α-chain oligomerization (Fig. 1). First, three α-chains oligomerize forming a triple-helical protomer. In mammals, three collagen IV protomers (α121, α345, and α565) are formed from six genetically distinct α-chains (α1–α6) (8). Second, two protomers oligomerize via their trimeric noncollagenous (NC1) domains forming an NC1 hexamer at the junction, and four protomers oligomerize via the 7S domain forming a tetrameric structure. Numerous studies have provided compelling evidence that the NC1 domain acts as a recognition module (8, 16, 17), specifying and directing the assembly of chain-specific scaffolds (Fig. 1).

In protomer oligomerization, the interaction between trimeric NC1 domains is a chloride-dependent event (16). The first evidence for a role of chloride ions in scaffold assembly was revealed from the crystal structure of α121 NC1 hexamers, derived from a native collagen IV scaffold by collagenase digestion, which showed the presence of a group of six Cl^−^ ions, three per NC1 trimer, embedded at the NC1 trimer–trimer interface (18). Our recent studies showed that the high concentration of Cl^−^ ions on the outside of cells signals protomer oligomerization and that Cl^−^ ions bound to NC1 trimers trigger a conformational switch, which mediates protomer oligomerization (16). Importantly, our recent studies of the crystal structures of α121 NC1 hexamers revealed an additional group of six Cl^−^ ions that are embedded at the trimer–trimer interface (19), indicating a role for these ions in hexamer assembly.

In this study, we investigated the mechanisms, kinetics, and functionality of the 12 chloride ions in protomer oligomerization in the formation of α121 scaffolds. For these studies, we used novel single-chain technology to produce a stable recombinant α121 NC1 trimer. We discovered that the 12 ions form a chloride ring at the interface of NC1 trimers, which induces assembly and stabilizes the NC1 hexamer. Collectively, our findings reveal a fundamental role for chloride ions in the assembly of collagen IV scaffolds of BMs, a critical event that enabled tissue evolution and development in animals.

## Results

### Experimental approach to investigate the role of chloride ions in hexamer assembly

In our previous study, we focused on the role of various ions in the assembly mechanism whereby NC1 monomers oligomerize forming NC1 hexamers. Chloride ions were found to induce hexamer assembly directly from monomers (16). Under these conditions, hexamer assembly involves two steps: monomers first oligomerize into trimers and, in turn, NC1 trimers oligomerize forming the NC1 hexamer. Initial findings suggest that chloride ions mediate the latter step, a critical step of scaffold assembly in tissues.

**Figure 2.**
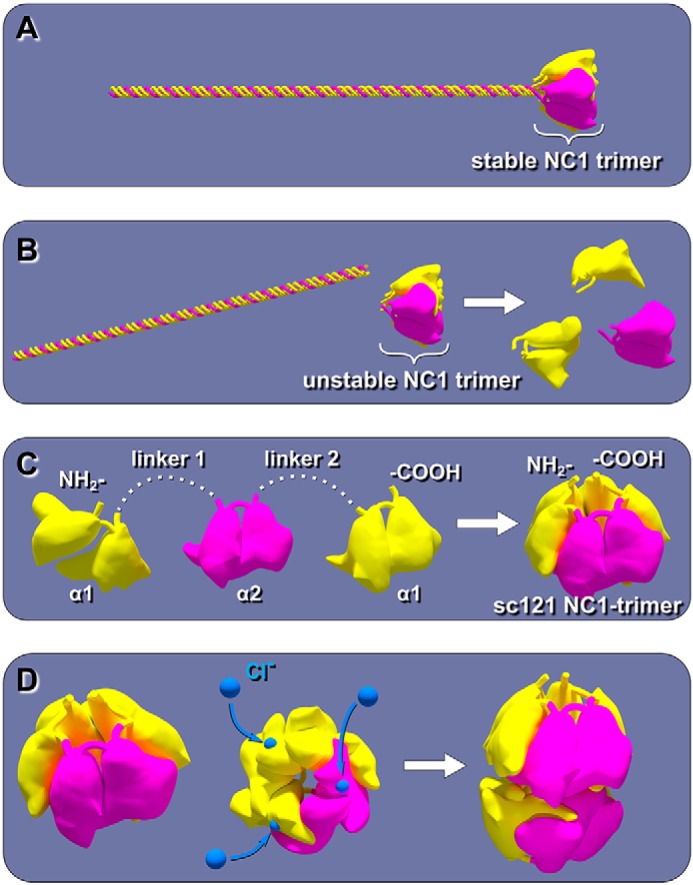
**Approach to generate a stable NC1 trimer without a triple helix.** *A,* in a collagen IV protomer, the intermediate state of the NC1 trimer is stabilized by the triple helix. *B,* without a triple-helical segment, the NC1 trimer dissociates into monomers under low-chloride concentration. *C,* using recombinant technology, a single-chain NC1-trimer composed of α1-, α2-, and α1-chains (sc121 NC1-trimer) can be generated. *D,* theoretically, sc121 NC1-trimer can be induced by chloride ions to self-assemble into a hexamer.

Here, we sought to directly investigate the mechanisms, kinetics, and function of chloride ions in the oligomerization of NC1 trimers forming hexamers (Fig. 2). A trimeric state of NC1 domains is only stable when it is attached to a folded triple helical portion of the collagen IV molecule (Fig. 2, *A* and *B*). Although a significantly shortened segment of triple helix can stabilize trimers of NC1 at low chloride concentrations (16), such trimers have limitations that precluded their use in studying the mechanism of NC1 trimer–to–hexamer assembly, a native process outside the cells. These limitations include a low yield in production, a susceptibility to proteolysis, and a loss of the trimeric structure at physiological temperature (16).

To overcome these challenges, we developed a novel strategy to stabilize the NC1 trimer. Analysis of the crystal structures of the α121 NC1 hexamer revealed that the N and C termini of adjacent domains of the NC1 trimer are proximal to each other (20, 21). Thus, using two linkers, three NC1 monomers can be linked into a single polypeptide (Fig. 2*C*). Furthermore, the sub-domain architecture of an NC1 monomer represents the shortest possible linker, which potentially would prevent undesired linker flexibility, add to trimer stability, provide better resistance to proteolysis of linkers, and exclude the possibility of domain swapping. Single-chain approaches have been successfully applied to produce and solve the crystal structures of trimerization domains of collagen-like proteins adiponectin (22) and complement protein C1q (23).

### Design of the single-chain α121 NC1 trimer

**Figure 3.**
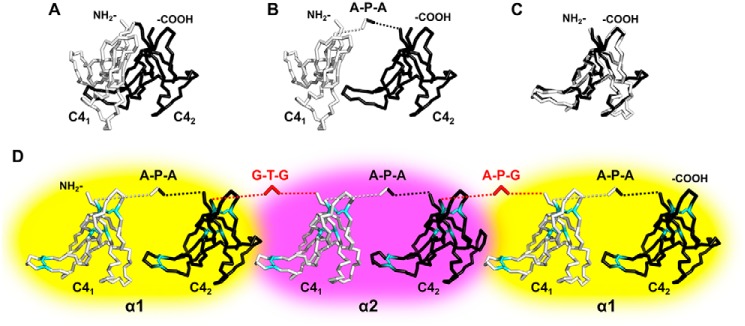
**Design of a single-chain NC1-trimer.** *A,* NC1 domain of each chain contains two C4 sub-domains. Shown are C4_1_ (*white*) and C4_2_ (*black*) sub-domains of the α1-chain in a backbone representation. *B,* two C4 sub-domains are pulled away to highlight a native three-residue linker Ala–Pro–Ala (*A-P-A*). *C,* trimmed C4_1_ and C4_2_ sub-domains are superimposed to demonstrate overall structural similarity and close geometrical positions of the N and C termini. *D,* single-chain construct design combining accordingly trimmed α1- (highlighted in *yellow*), α2- (*violet*), and α1 (*yellow*)-chains using similar–to–native linkers Gly–Thr–Gly (*G-T-G*) and Ala–Pro–Gly (*A-P-G*) (shown in *red*) between domains. Disulfide bonds are shown in *cyan* indicating their localization within the individual C4 sub-domains. The figure was generated using PDB code 1t61 of the collagen IV NC1 domain from the placenta basement membrane.

The monomeric NC1 domain of α1 and α2 collagen IV chains consists of two C4 sub-domains (C4_1_ and C4_2_) connected by a short three-residue linker (Fig. 3, *A* and *B*). The linker sequence is conserved with Ala-Pro-Ala being a consensus for α-chains of collagen IV (Fig. S1*A*). The C4 atomic structures are superimposable (Fig. 3*C*), and positions of either N or C termini trimmed down to core sequences (with removed linkers and terminal extensions) coincide. Thus, native C4_1_–C4_2_ connections can be supplemented with similar artificial connections between C4_2_ and C4_1_ of adjacent domains to generate a single-chain α121 NC1 trimer (sc121 NC1 trimer) combining six C4 sub-domains (Fig. 3*D*). Exact trimming and linking sequences for α1–α2 and α2–α1 connections are provided in Fig. S2, *B* and *C*. The resulting polypeptide construct (Fig. S2) also contains an N-terminal FLAG-tag sequence for immunoprecipitation and a signal peptide for secretion. Such a complex polypeptide should have a pathway for successful oxidative folding and formation of 18 disulfides. The polypeptide backbone of C4 has a knot-free topology, which does not require sequential folding of C4 sub-domains. We hypothesized that each C4 sub-domain represents an independent folding unit where the three disulfides (Fig. 3*D*) significantly restrain its structure upon oxidation. Indeed, the protein was expressed and secreted in a soluble form with all cysteines oxidized into disulfides (see below).

### sc121 NC1-trimers form NC1 hexamers in the presence of chloride ions

**Figure 4.**
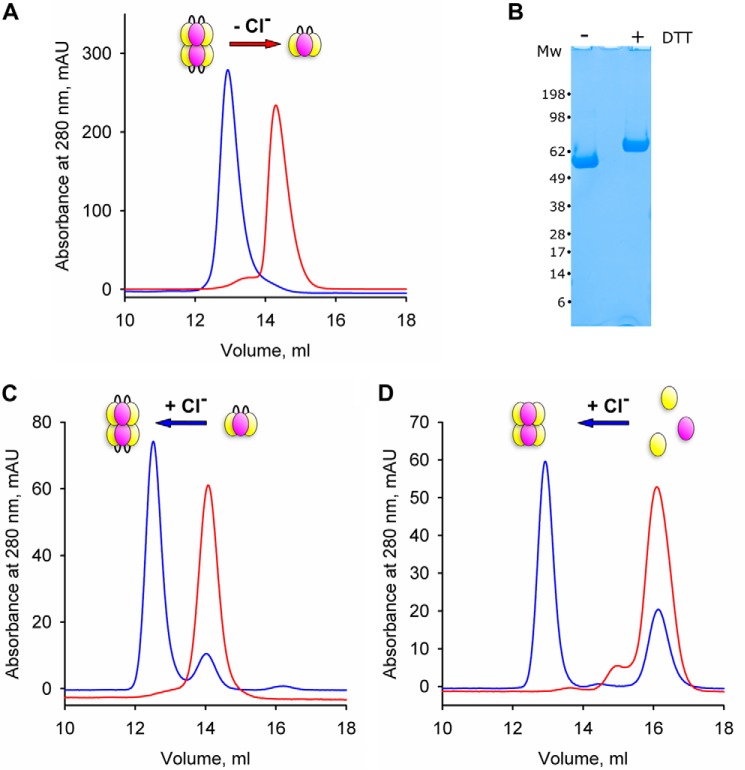
**Chloride controls dissociation and re-assembly of the NC1 hexamer.** *A,* sc121 NC1-trimer elutes from a size-exclusion column at two distinct positions depending on the presence (*blue*) or absence (*red*) of chloride, suggesting that it exists as a hexamer and a single-chain trimer, respectively. *B,* SDS-PAGE analysis of sc121 NC1-trimer under nonreducing and reducing conditions demonstrates homogeneity of the protein and the presence of intramolecular disulfide bonds. *C,* chloride ions induce re-assembly of the hexamer from sc121 NC1-trimers. *D,* similar re-assembly of the hexamer from NC1 domain monomers purified from bovine lens basement membrane occurs under the same conditions.

The sc121 NC1-trimer was collected from the cell culture medium containing a regular Cl^−^ concentration and purified in the presence of 150 mm NaCl. After size-exclusion chromatography (SEC) on a column equilibrated with 25 mm Tris-HCl, pH 7.5, supplemented with 150 mm NaCl, the protein eluted as a single peak (Fig. 4*A*, indicated by *blue line*) with an apparent molecular mass of 113 kDa, according to a protein calibration kit. The major peak was pooled and analyzed by SDS-PAGE, which showed a single band that was sensitive to reduction (Fig. 4*B*), expected because the protein contains 18 potential disulfide bonds. The protein was concentrated to the same volume of sample that was initially applied to the column, and was re-run over the same column, but equilibrated with Cl^−^-free buffer, 25 mm Tris acetate, pH 7.5, supplemented with 150 mm sodium acetate. A clear shift was detected for the position of the major peak with an elution volume that corresponded to an apparent molecular mass of 60 kDa (Fig. 4*A*, indicated by *red line*).

These results suggest that the sc121 NC1-trimer occurred in a hexamer configuration in the cell culture medium, but upon removal of chloride the hexamer dissociated into NC1 trimers. The apparent molecular masses are less than expected, as the calculated molecular mass of the single-chain NC1-trimer is 76.2 kDa and the corresponding hexamer is 152.4 kDa. This phenomenon is consistent with our previous data on chloride-dependent hexamer assembly using monomeric NC1 chains (16). Whereas elution profiles of high molecular mass complexes assembled from monomeric or single-chain trimeric NC1 domains are identical on a SEC column in the presence of chloride, the samples in Cl^−^-free buffer eluted at different positions, corresponding to the monomeric and trimeric states (Fig. 4, *C* and *D*, indicated by *red lines*). Upon the addition of chloride, sc121 NC1-trimers re-assembled into hexamers (Fig. 4*C*). This behavior is identical to that of NC1 monomers in the presence of chloride, as described previously (Fig. 4*D*) (16).

Moreover, sc121 NC1-trimers have the capacity to co-assemble with tissue-derived α1 and α2 NC1 monomers, forming a complex that elutes in the position of NC1 hexamer. The monomers were incubated with the sc121 NC1-trimer in the presence of Cl^−^, and a composition of formed hexamers was analyzed after immunoprecipitation with the FLAG tag of the recombinant trimer (Fig. S3). Both bovine α1 and α2 NC1 monomers were incorporated into the heterotypic hexamer along with the sc121 NC1-trimer. Together, these results indicate that the sc121 NC1-trimer harbors the capacity to interact with chloride ions which induces (*a*) self-dimerization forming a hexamer and (*b*) oligomerization with NC1 monomers forming a heterotypic hexamer.

### Atomic model of NC1 hexamer assembled from sc121 NC1-trimers

**Figure 5.**
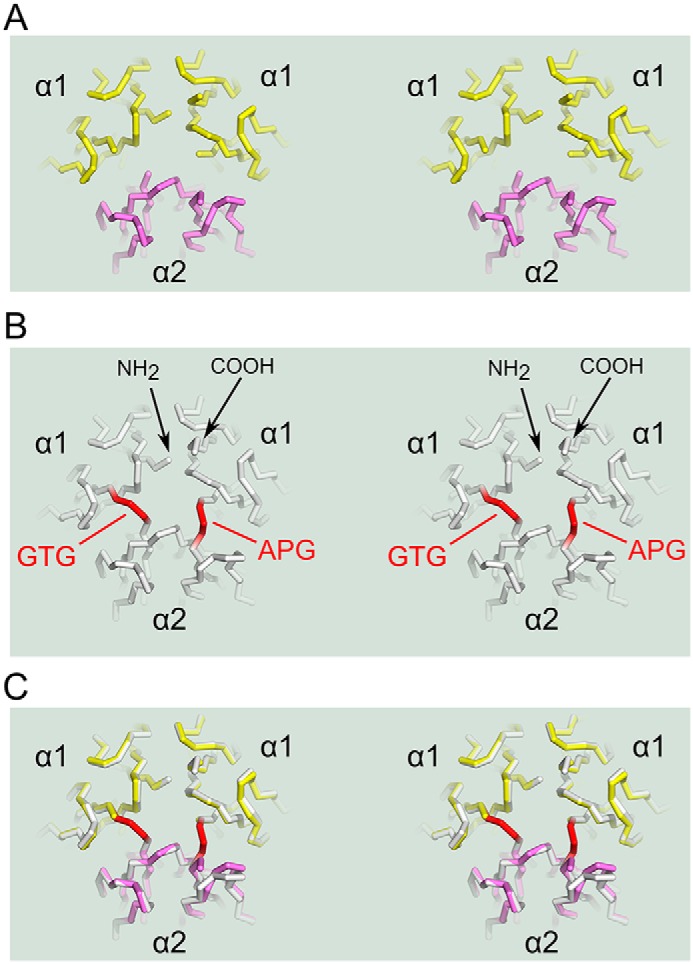
**Artificial linkers mimic the pattern of native C4–C4 connections.** Stereo pairs of backbone wire-frames are viewed from putative triple-helical part for native NC1-trimer (*A*) or sc121 NC1-trimer (*B*). Two linkers, Gly-Thr-Gly (*GTG*) and Ala-Pro-Gly (*APG*) (shown in *red*), connect three chains into a single polypeptide. *C,* superimposition of native and sc121 NC1-trimers shows minimal distortion from introduced linkers.

The putative NC1 hexamer formed from sc121 NC1-trimers in the presence of chloride was collected from an SEC column, concentrated, and used for crystallization trials while maintaining the high chloride concentration. Ultimately, it was crystallized in space group P4_1_2_1_2 with a single polypeptide chain per asymmetric unit (Table S1). The crystal structure was determined using X-ray diffraction. The overall atomic structure is identical to the previously reported structures for human and bovine NC1 domains isolated from tissues (Fig. S4). Least-square superimpositions revealed no significant variations between corresponding Cα atoms. Despite being of human origin, our structure fits bovine structures (1t60 and 1t61, RMSD 0.35–0.38 Å) slightly better than human (PDB code 1LI1, RMSD ∼0.49 Å). These minor discrepancies can be attributed to the different crystallization conditions and crystal packing. Structural identity for native and recombinant proteins demonstrates the validity of our strategy of sc121 NC1-trimer production. All C4–C4 linkers, native and artificial, are well-structured and are related by a pseudo-hexagonal symmetry (Fig. 5). All linker residues have a well-defined electron density map (Fig. S5). Although slightly higher for the artificial sequences, atomic displacement factors are comparable (Fig. S5*F*).

**Figure 6.**
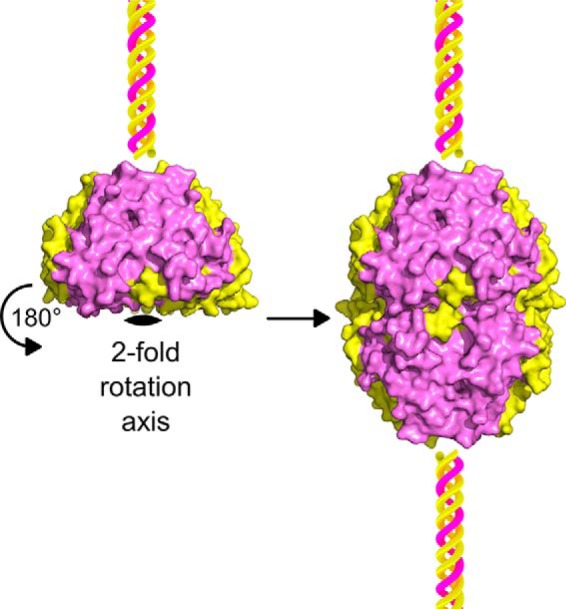
**Two single-chain NC1-trimers form a hexamer.** Asymmetric unit is a single polypeptide chain of the sc121 NC1-trimer represented as a surface marked in *yellow* for α1 and *violet* for α2 NC1 domains. A triple helix, which is not a part of this structure, is shown as a *cartoon* for orientation purpose. The biological unit of a hexamer observed in the crystal structure can be generated by applying a crystallographic 2-fold rotation symmetry to the asymmetric unit.

Previously reported structures of NC1 domains derived from α121 scaffolds have one or several hexamers per asymmetric unit, where each hexamer contains two NC1 trimers related by a pseudo 2-fold rotation symmetry. Here, the hexamer is formed by two sc121 NC1-trimers related by a crystal 2-fold rotation symmetry (Fig. 6). These results show that in the presence of chloride the sc121 NC1-trimer assembles into a hexamer configuration.

### Two groups of chloride ions at the hexamer interface

In the crystal structure of α121 NC1 hexamers, derived from a native collagen IV scaffold, two sets of ions were found at the interface between NC1 trimers and assigned to six Cl^−^ and six K^+^ ions (18). Our recent data on NC1 hexamer assembly clearly demonstrated a Cl^−^- but not K^+^-dependent mechanism of assembly (16), prompting us to reassess the identity of K^+^ ions. Re-analysis of structural data pointed to a possibility that sites originally assigned to K^+^ are rather occupied by an additional six Cl^−^. Very recently, the same conclusion was reached based on structural data of different NC1 hexamers assembled from recombinant polypeptides (19). The number of ions at the trimer–trimer interface of α121 NC1 hexamers in reported structures varies depending on protein source, preparation, crystallization conditions, and resolution, where 6, 8, 11, 12, and 14 ions per hexamer were identified (18–21). To determine the number and positions of Cl^−^ ions involved into stabilization of the NC1 hexamer, here we purposely purified and crystallized the sc121 NC1-trimer in the presence of high chloride concentration to mimic natural extracellular Cl^−^ content.

**Figure 7.**
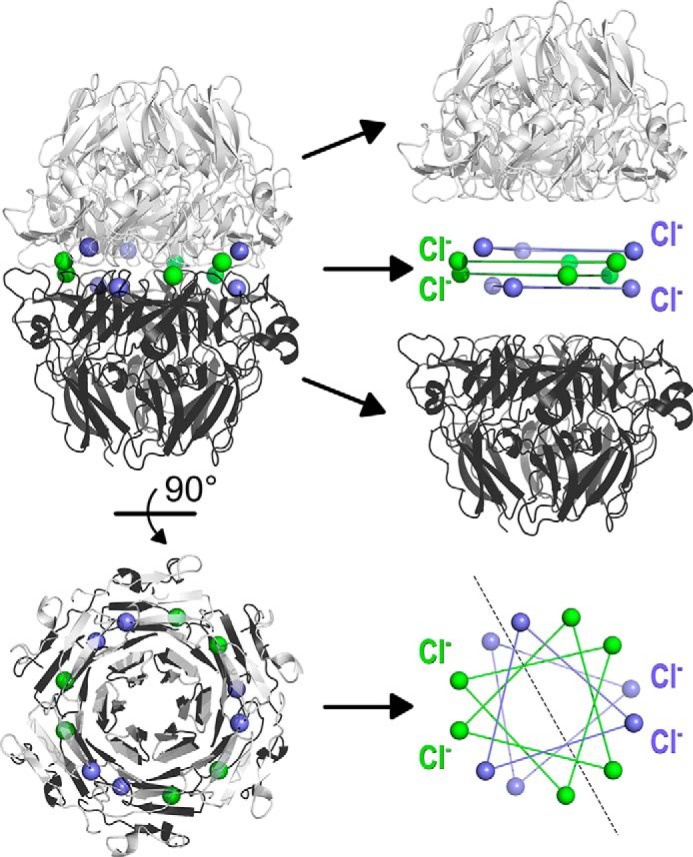
**Two groups of chloride ions coordinate the hexamer structure composed of two sc121 NC1-trimers.** The biological unit, a hexamer, is depicted as *ribbons*. Interaction of two sc121 NC1-trimers (shown in *light gray* and *black*) is mediated by two groups of chloride ions (shown in *blue* for group 1 and *green* for group 2). Each group has two symmetry-related layers. All four layers are parallel to each other and to the equatorial plane of the hexamer. *Solid lines* are drawn between Cl^−^ ions for each layer for presenting an array of planes. A *dashed line* represents a 2-fold rotation symmetry axis.

**Figure 8.**
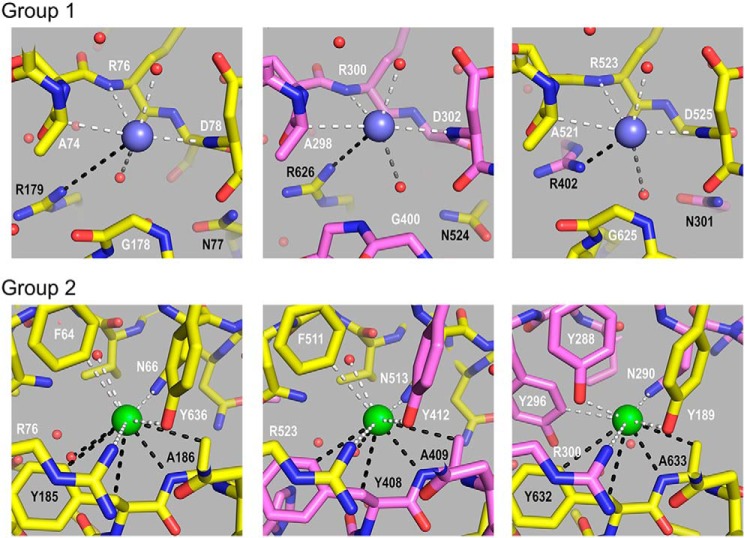
**Chloride ions coordinate the sc121 NC1-trimer–trimer interface.** Interactions for group 1 (*blue spheres*) and group 2 (*green*) Cl^−^ ions are shown. Residues of the one sc121 NC1-trimer are labeled in *white* and of the opposite sc121 NC1-trimer is in *black*. Coordination by residues and water molecules is shown as *white* and *black dashed lines* for the two trimers, respectively. Additional water molecules participate in coordination of group 1 chloride ions, as shown by *gray dashed lines* (*upper panel*).

Analysis of the hexamer formed from sc121 NC1-trimers revealed a set of 12 Cl^−^ ions at the trimer–trimer interface (Fig. 7). These ions form two structurally different groups (Fig. 8). Group 1 has been previously identified (18) and includes six chloride ions (16). Group 2 has been recently suggested and also includes six chloride ions (19). Several factors support the Cl^−^ nature for the observed electron densities (Fig. S6). Original phasing for the NC1 domain was done using an anomalous signal from Br^−^ ions (belonging to the same halide group as Cl^−^). All 12 positions (both groups) exhibited anomalous signals from bromide ions. Although crystal soaking was done using KBr salt, these sites were occupied by Br^−^ and not K^+^ ions. A crystal structure of the NC1 domain derived from human placenta contains six acetate ions instead of group 2 chlorides (chloride ions were excluded during protein preparation and in crystallization solutions) indicating the preference for negative charges at these locations. Collectively, all 12 Cl^−^ at the trimer–trimer interface have well-defined and comparable electron density (Fig. S6) and B-factor values in the range from 23 to 27 Å^2^, like atoms of nearby residues. Together, the 12 ions form a chloride ring at the hexamer interface (Fig. 7).

**Figure 9.**
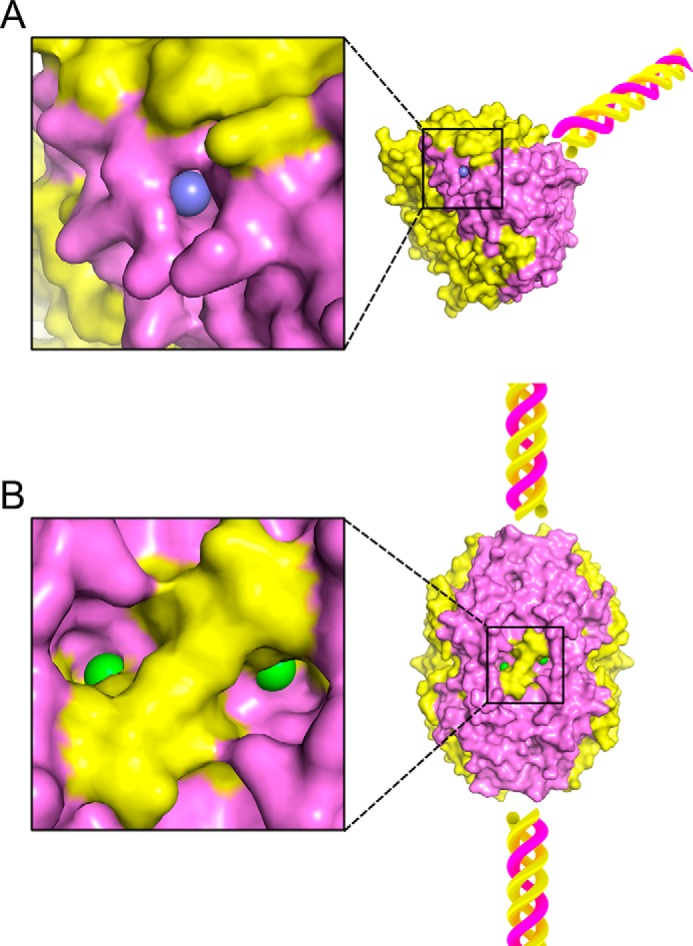
**Solvent accessibility of groups 1 and 2 chloride ions.** Group 1 ions (*blue*) are solvent-accessible in the sc121 NC1-trimer (*A*) but not in the assembled hexamer. In contrast, group 2 ions (*green*) are solvent-accessible in the assembled hexamer through portals (*B*). Solvent-accessible surfaces of the trimer and the hexamer are colored in *yellow* for α1 and in *violet* for α2. Fragments of the triple helix are shown as *cartoons* for orientation purposes only and are not part of the reported structure.

Analysis of the solvent-accessible surface of the trimer and the hexamer shows that group 1 chloride ions are solvent-accessible in the trimer only, but group 2 ions remain solvent-exposed in the assembled hexamer (Fig. 9). Every Cl^−^ of group 2 is sitting in a pocket, which communicates with the outside through a portal. The nature and geometry of such a structure points to a sensing mechanism of chloride concentration, where bound Cl^−^ ions are in dynamic equilibrium with free ions in solution.

### Characterization of the oligomeric states of sc121 NC1-trimer in solution

**Figure 10.**
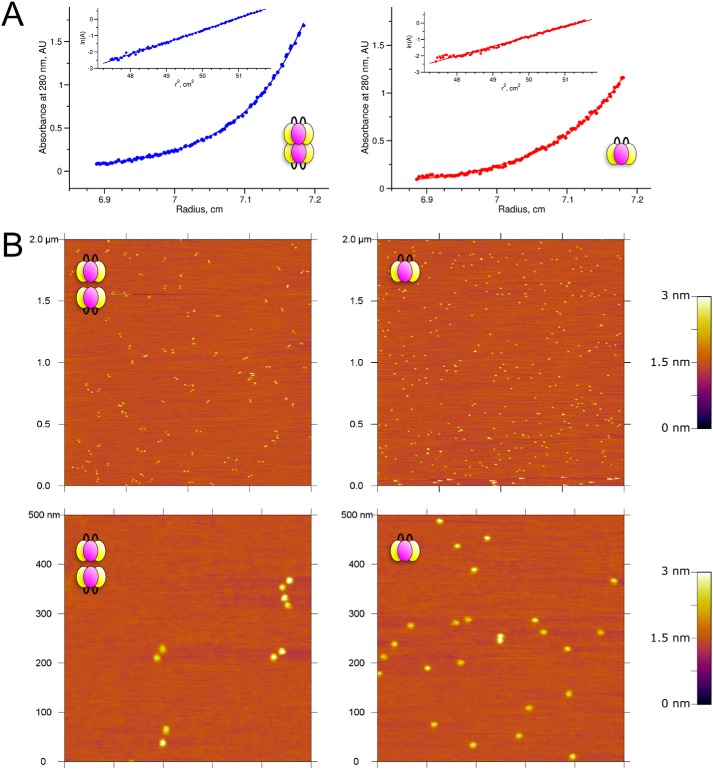
**Oligomeric state of sc121 NC1-trimer under Cl^−^-containing and Cl^−^-free conditions.** *A,* sedimentation equilibrium experiments using analytical centrifugation demonstrate the difference in molecular mass of the sc121 NC1-trimer in Cl^−^-containing (*blue*) and Cl^−^-free (*red*) buffers. *B,* atomic force microscopy images of the same samples are used to directly visualize the difference in oligomeric state of the sc121 NC1-trimer. *Top,* wide-field; *bottom,* closer view. When deposited from Cl^−^-containing buffers (*left*), sc121-NC1 initially associated with the mica as hexamers, rinsing with water-induced dissociation into pairs of trimers. Conversely, only randomly distributed sc121 NC1-trimers were observed when deposited from Cl^−^-free solutions (*right*).

To verify oligomeric states in solution, we performed sedimentation equilibrium ultracentrifugation (Fig. 10*A*). For material collected from the major peak in the Cl^−^-rich conditions, we determined the molecular mass of 148 ± 10 kDa, which is consistent with the hexameric state of NC1 (calculated mass is 152 kDa). Cl^−^-free material demonstrated a nonideal behavior and an average molecular mass of 100 ± 8 kDa (expected mass for the trimer is 76 kDa), which might reflect an equilibrium between a hexamer and a trimer. Indeed, using a model of trimer–to–hexamer self-association with the mass of the trimer fixed to 74 kDa, we fitted the sedimentation equilibrium curve and estimated the *K_d_* value to be 37 μm. The fraction of trimer under the experimental conditions (0.3 mg/ml concentration of the protein in Cl^−^-free buffer) corresponded to ∼89%. Because the hexamer fraction was not observed on the sieve column, the *k*_off_ of the hexamer–to–trimer reaction is significantly faster than running time of a size-exclusion chromatography (∼30 min).

We verified the Cl^−^-dependent oligomeric state of our samples using an imaging mode of atomic force microscopy (AFM) (Fig. 10*B*). Sample preparation included incubation of 20–40 μl containing tens of nanograms of protein at the surface of freshly cleaved mica to allow for noncovalent adhesion of molecules followed by a short wash with deionized water and fast drying. The wash step with water removed Cl^−^ and thus initiated dissociation of the hexamers into sc121 NC1-trimers. Surprisingly, dissociated molecules stayed in contact with mica while diffused on the surface (Fig. 10*B*). As a result, we observed pairs of NC1 trimers for Cl^−^-containing samples and random distribution of NC1 trimers prepared from Cl^−^-free solutions.

**Figure 11.**
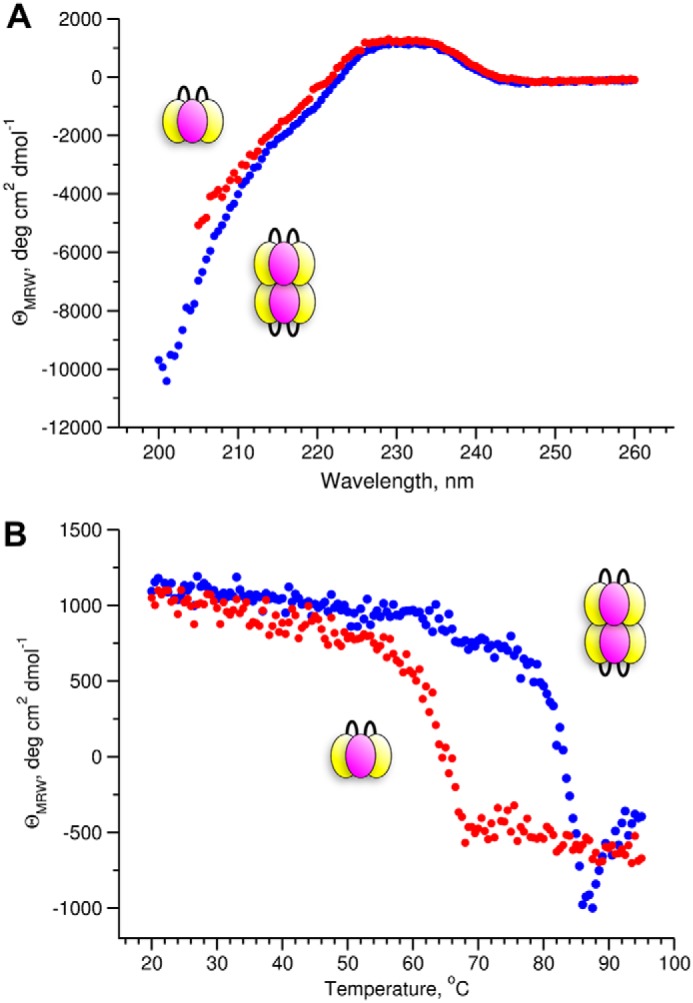
**Chloride has a minor effect on secondary structure, but thermal stability of the hexamer is greatly increased compared with the trimer.** *A,* CD spectra of the NC1 hexamer assembled from sc121 NC1-trimers in Cl^−^-containing buffer (*blue*) and in Cl^−^-free buffer (*red*). *B,* thermal denaturation profile was monitored by measuring the CD signal at 230 nm.

CD far-UV spectrum (Fig. 11*A*) confirmed the identity of the secondary structure content of the recombinant single-chain NC1 domain and tissue-derived NC1 domains (24). Moreover, this content is largely the same for the hexamer and the trimer. However, the hexamer stabilized by the Cl^−^ ions demonstrated significantly higher (by ∼20 °C) resistance to irreversible heat denaturation and precipitation than the trimer in the Cl^−^-free buffer (Fig. 11*B*).

Together, our results confirmed the Cl^−^-dependent nature of the NC1 hexamer and competence of our artificially stabilized single chain sc121 NC1-trimer for hexamer assembly. The trimer at low Cl^−^ concentration represents an intermediate state suitable for quantitative analysis of the hexamer assembly. This system provides an excellent model for studying the process of hexamer assembly induced by exposure to high Cl^−^ concentration, which happens outside cells when full-length collagen IV is secreted to the extracellular environment.

### Kinetics of hexamer assembly from sc121 NC1-trimers

**Figure 12.**
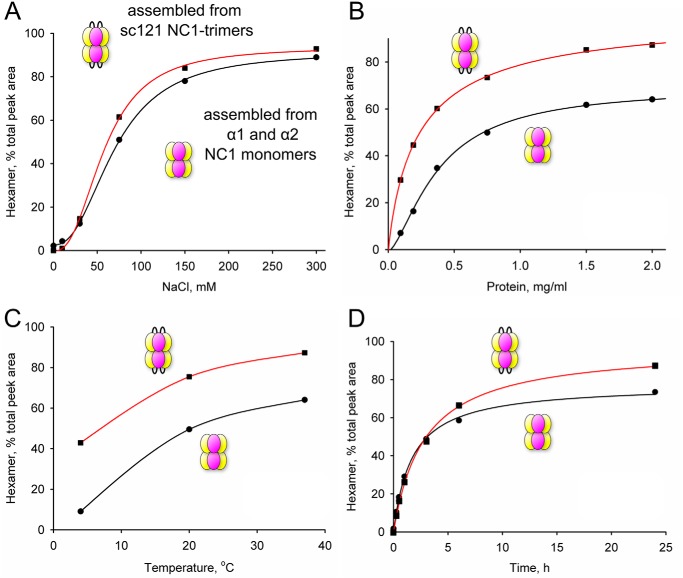
**sc121 NC1-trimer is more efficient for hexamer assembly than NC1 monomers.** The assembly assay was performed by varying one of the following parameters: NaCl (*A*) or protein (*B*) concentration and temperature (*C*) or incubation time (*D*). Size-exclusion chromatography was used to quantitate hexamer assembly as a percentage of the total peak area. Experimental data (*dots*) were fitted using four-parameter logistic, hyperbolic, or cubic spline for presentation purposes only.

We quantitatively analyzed the assembly process of the hexamer from the NC1 intermediate-state trimer under varying conditions. We performed a series of experiments under different Cl^−^ concentrations, protein concentrations, and temperature and measured the assembly kinetics (Fig. 12) using the sc121 NC1-trimer as well as monomers for comparison. For all conditions, the sc121 NC1-trimer assembled into the hexamer more efficiently than monomers, except very low Cl^−^ concentrations (Fig. 12*A*) and initial kinetics (Fig. 12*D*), where the sc121 NC1-trimer behaved indistinguishably from monomers. Trimer–to–hexamer assembly should follow a simple bimolecular reaction mechanism (Reaction 1) where two trimers T form a hexamer H, as opposed to a multistep assembly process from monomers M.

$$\begin{array}{c} \text{T}+\text{T}⇄\text{H}\\ \text{Reaction}\;1 \end{array}$$

Two constants, *k_a_* and *k_d_*, describe association and dissociation rates, respectively, via the differential Equation 1.

(Eq. 1) $$\frac{\text{d}T}{\text{d}t}=-k_{a}{\text{T}}^{2}+k_{d}\text{H}=-2\frac{\text{dH}}{\text{d}t}$$

In case of high Cl^−^ concentration, *k_d_* is negligible (the hexamer is stable), and the reaction can be described by a single rate constant *k_a_*.

(Eq. 2) $$\text{T}+\text{T}\overset{k_{a}}{\to }\text{H}$$

The differential Equation 2 becomes Equation 3.

(Eq. 3) $$\frac{\text{d}\left[T\right]}{\text{d}t}=-k_{a}\cdot {\left[\text{T}\right]}^{2}$$

Its general solution is given by Equation 4.

(Eq. 4) $$\left[\text{T}\right]=\frac{1}{k_{a}\cdot t+\text{const}}$$

Given that all protein is trimeric at time point *t* = 0, the final solution is shown in Equation 5.

(Eq. 5) $$\left[\text{T}\right]=\frac{1}{k_{a}\cdot t+\frac{1}{T_{0}}}$$

This can be used to find the time-dependent concentration of the hexamer as shown in Equation 6.

(Eq. 6) $$\left[\text{H}\right]=\frac{T_{0}-\left[\text{T}\right]}{2}=\frac{T_{0}}{2}-\frac{1}{2k_{a}\cdot t+\frac{2}{T_{0}}}$$

We used Equation 6 to fit experimental kinetic data (Fig. S7*A*) and derived *k_a_* = 3.45 ± 0.12 m^−1^ s^−1^. Equation 6 allows us to predict the hexamer fraction at any given time and from any starting trimer concentration. Predicted concentration-dependent data perfectly fit the experimental values (Fig. S7*B*), further validating the second-order reaction model. As expected, the kinetics are concentration-dependent. Theoretical values calculated for the hexamer formation kinetics are presented in Fig. S7*C* using a range of protein concentrations.

To estimate the activation energy *E_a_* for assembly, we fit the temperature data (Fig. S7*D*) using Equation 6 and the following form of the Arrhenius Equation 7 for *k*_*a*_^*T*^,

(Eq. 7) $$k_{a}^{T}=k_{a}^{T_{0}}e^{-E_{a}\left(\frac{1}{RT}-\frac{1}{RT_{0}}\right)}$$

where *k*_*a*_^*T*_0_^ = *k_a_* at *T*_0_ = 310.15 K (37 °C). The resulting value of the activation energy is 49.5 ± 2.3 kJ m^−1^.

### Evolution of the chloride ring

**Figure 13.**
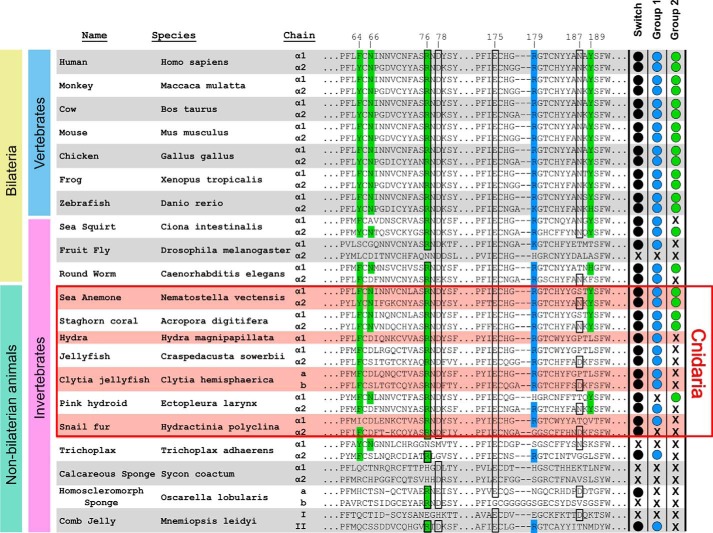
**Evolutionary analysis of amino acid residues of collagen IV NC1 domains that are directly involved in chloride-dependent hexamer assembly.** The side chain of Arg/Lys-179 (*blue*) is coordinated by a group 1 chloride ion of the opposite trimer. Group 2 chloride ions coordinate side chains of residues Phe/Tyr-64, Asn-66, Arg-76, and Tyr/His-189 of both trimers (*green*). Chloride ions of group 1 are also involved in switching of Arg-76 interaction with Asp-78 on the same trimer to Glu-175 and Asn/Asp-187 on the opposite trimer (*all boxed*) (16). The chloride switch is determined by the presence of at least residues Arg-76, Asp/Glu-78, and Glu-175. Prediction of group 1 Cl^−^ coordination (*blue ball*) is based on the presence of Arg/Lys-179 directly involved in binding to the ion. Prediction of group 2 Cl^−^ coordination (*green ball*) is based on the presence of all four residues, Phe/Tyr-64, Asn-66, Arg-76, and Tyr/His-189.

Collagen IV is conserved across all animals (1, 10); however, the evolutionary conservation of the chloride ring is unknown. To determine the evolutionary origin and conservation of the chloride ring and the residues of the chloride switch (16), we aligned amino acid sequences of NC1 domains across several metazoan representatives and annotated those residues whose side chains coordinate chloride ions and participate in the chloride switch (Fig. 13). Group 1 residues occur in at least one α-chain in all animals, except for both sponge representatives. Group 2 residues first occurred within cnidarians and were conserved across bilaterians, except for fruit fly and one collagen IV chain in *Caenorhabditis elegans* and *Ciona intestinalis*.

Residues of the chloride switch occur in at least one α-chain in all species, except for the calcareous sponge representative (Fig. 13). Interestingly, in fruit fly, the α2-chain lacks the switch, group 1, and group 2 residues, whereas the α1-chain contains switch and group 1 residues, suggesting an alternative mechanism of hexamer assembly. Importantly, there is a transition within nonbilaterian animals wherein only switch and group 1 residues occur in *Trichoplax*, sponges, and comb jelly, whereas the switch, group 1 and group 2 residues occur within cnidarians and were conserved across bilaterians. This analysis indicates that the chloride ring is a fundamental feature of collagen IV scaffolds that first appeared in a last common ancestor of cnidarians and bilaterians.

## Discussion

**Figure 14.**
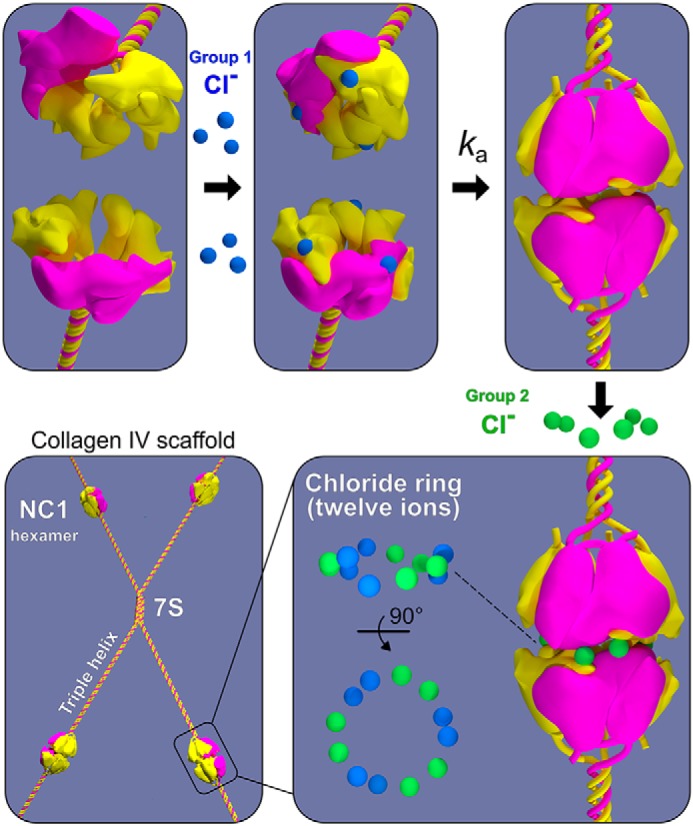
**Summary model for the role of the chloride ring in the assembly of collagen IV scaffolds.** Group 1 Cl^−^ ions, three per trimer, populate two NC1 trimers, activating the surface for docking and hexamer formation. The bimolecular assembly reaction proceeds with the *k_a_* rate constant. Finally, the quaternary structure of the hexamer is stabilized by six group 2 Cl^−^ ions, three per trimer. The chloride ring of 12 remains a structural component providing integrity to the collagen IV scaffold.

BM signaling is pivotal to cell behavior and differentiation, yet many assembly mechanisms of its toolkit proteins remain undefined (7, 25), including those for collagen IV scaffolds (5, 26). Collagen secretion is accompanied by a change of chloride concentration from about 5 mm inside the cells to about 100 mm on the outside of the cells. Such a chloride concentration switch triggers the formation of the NC1 hexamer, a critical step in the assembly of collagen IV scaffold (16). The single-chain NC1-trimer technology provided an approach to directly investigate how chloride ions induce collagen IV protomer oligomerization (Fig. 2), a key step in scaffold assembly (5, 16, 26). We found that 12 chloride ions form a chloride ring at the hexamer interface (Fig. 7) and induce the sc121 NC1-trimer to self-assemble (Fig. 4) into hexamer with an atomic structure identical to that of hexamers from tissues (Fig. S4) (18, 21). The chloride ring is composed of two structurally distinct groups of six ions. Group 1 ions induce hexamer assembly, and group 2 ions stabilize the hexamer structure. The overall mechanism of chloride function in collagen IV scaffold assembly in a biological context is summarized in Fig. 14.

In the first stage of oligomerization, group 1 ions activate a molecular switch within the NC1-trimeric domain that induces trimer–trimer interactions, forming an NC1 hexamer (Fig. 14), as shown previously (16). Extracellular Cl^−^ disrupts the intramolecular salt bridge Arg-76–Asp-78, and specific binding of a Cl^−^ ion causes coordination of the Arg-76 backbone amide, thus orienting the side chain toward an opposing NC1 trimer and ultimately forming salt bridges with Glu-175 and Asn-187 side chains (16). Cl^−^ ion coordination is fulfilled by a short loop (residues α1_74–78, α2_298–302, or α1_521–525), ionic interaction with arginine side chain (α1_Arg-179, α1_Arg-626, or α2_Arg-402) of an opposing trimer, and hydrogen bonding with two water molecules (Fig. 8 and Table S2). Within the hexamer structure, group 1 ions are buried deep and are located on the trimer face, hooked by the backbone loops that provide two amide and one Cα hydrogen bonds (Fig. 8).

In the second stage of oligomerization, group 2 ions stabilize the quaternary structure of the assembled hexamer (Fig. 14). Each ion has extensive hydrogen bonding from both trimers and ionic interaction with the side chain of arginine (α1_Arg-76, α1_Arg-523, or α2_Arg-300) (Fig. 8 and Table S2). Interestingly, these arginines are re-oriented by group 1 chlorides (16). Thus, group 1 and group 2 ions form a continuous network of interactions that stabilize the hexamer interface. Sequence variations between α1- and α2-chains lead to two patterns of coordination, with or without water molecules. In the α2-chain, the hydroxyl group of Tyr-288 (Phe-64 and Phe-511 in α1) coordinates Cl^−^ instead of one water molecule, and the side chain of Tyr-296 (Asn-183 and Asn-630 in α1) provides C–H hydrogen bonding and sterically excludes the possibility of accommodating another water molecule (Fig. 8 and Table S2). Collectively, each Cl^−^ ion of group 2 coordinates three O–H (two for water-free pattern), two N–H, and four C–H hydrogen bonds and one ionic interaction (Table S2).

Group 2 Cl^−^ ions, located close to the equatorial plane of the hexamer (Fig. 7), are probably in equilibrium with solution, so local Cl^−^ concentration is crucial for maintaining the population of these sites and hexamer stability. The mechanism of assembly and stabilization is fully reversible (Fig. 4). Exchange of chloride ions from the medium to the binding sites can proceed through open portals (Fig. 8*B*). If Cl^−^ concentration is insufficient to maintain a critical number of group 2 ions, the hexamer will dissociate into trimers where group 1 ions will become solvent-accessible and lost, which would consequently lead to structural changes at the trimer interface making it incompatible with trimer–trimer docking. In summary, the 12 Cl^−^ ions of groups 1 and 2 constitute a chloride ring located at the trimer–trimer interface of the hexamer. Functionally, group 1 ions activate the NC1 trimer to assemble into an NC1 hexamer, and group 2 ions stabilize the hexamer structure (Fig. 14).

Importantly, the chloride ring was an evolutionarily ancient innovation that is highly conserved across the animal kingdom (Fig. 13). Binding sites for group 1 ions and residues involved with the chloride switch emerged first in the last common ancestor of nonbilaterian animals and were conserved throughout the animal kingdom. Binding sites for group 2 ions emerged in the last common ancestor of cnidarians and bilaterians and were conserved throughout Bilateria. Thus, we conclude that the chloride ring is a fundamental structural feature mediating the assembly of collagen IV scaffolds of basement membranes and represents a critical innovation that helped enable tissue evolution and development.

A growing number of examples indicate that the chloride gradient between intracellular compartments and the extracellular environment has a general impact on secreted proteins. In addition to collagen IV assembly (16), the gradient is critical for collagen type I fibril formation (27, 28). Another example is the direct effect of chloride on WNK1 kinase signaling (29). Low Cl^−^ concentration inside cells prevents premature network formation of ECM molecules (16) and may help to minimize the size of molecules for secretion (30). Similar to our findings, chloride can affect oligomerization of other proteins as has been shown for the dimerization of bacterial alkaline phosphatase (31). Moreover, Cl^−^ is a significant hydrogen bond acceptor that can coordinate multiple groups at the same time. Given the abundance of Cl^−^ in all organisms, the structural and functional impact of this ion is probably underappreciated.

Chloride ion is vital for multiple physiological processes. Disbalance of chloride concentration affects blood pressure, gas exchange, acid-base equilibrium, neural signal transduction, gastrointestinal and kidney functions, etc. (32). A dramatic decrease, by about 30%, of chloride concentration in blood plasma is observed in patients with cystic fibrosis (33). All these conditions might also lead to defects in the basement membrane due to impaired collagen IV scaffold formation, a new aspect to be investigated.

Finally, we conclude that the single-chain NC1-trimer is a novel and powerful tool to study a plethora of questions about the mechanisms underlying specificity and stability of the NC1 trimer and hexamer assembly. The rationale for this is competence of the sc121 NC1-trimer for Cl^−^-dependent self-assembly of hexamer, the structural identity of the hexamer to the native structure, and co-assembly of sc121 NC1-trimer with tissue-derived NC1 monomers forming a heterotypic hexamer. Moreover, this single-chain trimer technology can be used to explore assembly, structure, and function of α121, α345, and α565 collagen IV scaffolds, as well as the pathogenesis of diseases involving these scaffolds. In addition, the technology provides a framework for production, formulation, and administration of potential collagen IV replacement therapies in Alport syndrome, and for screening of drug candidates as inhibitors of scaffold assembly for use as anti-angiogenic and anti-fibrotic agents.

## Experimental procedures

### Cloning, expression, and purification

Human sequences encoding residues 1438–1666 of α1-chain, a Gly–Thr–Gly linker, residues 1490–1709 of α2-chain, a Ala–Pro–Gly linker, and residues 1446–1669 (including final C-terminal residues) of α1-chain of collagen IV NC1 domain were subsequently cloned in-frame with the SPARC signal peptide and the FLAG tag of the pRc-X vector (34) (pRc-X_α1α2α1–scNC1) between restriction sites NheI and BspDI. Linkers between α1–α2-chains and α2–α1-chains were designed to contain restriction sites KpnI and XmaI, respectively (encoding Gly-Thr and Pro-Gly residues of Gly-Thr-Gly and Ala-Pro-Gly linkers). These restriction sites simplified cloning and generated a convenient vector suitable for generation of other combinations of collagen IV chains. The DNA sequence is available upon request. The resulting plasmid was sequence-verified and transfected into HEK293 cells. Stable clones were isolated using antibiotic selection with G-418. Protein expression in conditioned media was verified by Western blotting using rat anti-collagen IV NC1 (1:250 dilution, JK2; from Y. Sado, Shigei Medical Research Institute, Okayama, Japan). Several clones were selected based on the highest level of expression of sc121 NC1-trimer. The recombinant protein fused with an N-terminal FLAG-tag was purified as described (35). Size-exclusion chromatography using Superdex 200 Increase 10/300 GL column (GE Healthcare) was used for the final purification steps. Purified product yielded a single band on SDS-PAGE under nonreducing conditions verifying homogeneous oxidation of disulfide bonds (Fig. 9*B*). The final yield from two selected clones varied from 1.5 to 3 mg/liter of serum-free Dulbecco's modified Eagle's medium to 3–6 mg/liter of media supplemented with 10% fetal bovine serum.

### Crystallization and structure determination

The sc121 NC1-trimer was crystallized in tetragonal form (space group, P4_1_2_1_2_1_) using the hanging drop vapor diffusion method. The protein solution (11 mg/ml) in 5 mm Tris-HCl, pH 7.5, 150 mm NaCl was mixed for the drop solution in a 1:1 proportion with a reservoir solution of 0.085 m HEPES, pH 7.5, 1.68 m ammonium sulfate, and 1.5% (w/v) PEG 400. The crystals grew to a final size of 0.5 × 0.5 × 0.3 mm after 2–5 days at 22 °C. The crystals were briefly dipped into a cryoprotectant solution containing the reservoir solution and 30% (v/v) glycerol and then frozen in liquid nitrogen. Data collection was performed remotely on crystals cryocooled to 100 K at the Life Sciences Collaborative Access Team beamline 21-ID-G at the Advanced Photon Source, Argonne National Laboratory. Data extending to 1.9 Å resolution were indexed using iMOSFLM (36) and then scaled and merged using Scala (37). Amplitudes were converted to structure factors using Ctruncate (38). Five percent of the data were set aside to monitor *R*_free_. Initial phases were obtained by molecular replacement using Phaser-MR (39) and the previously solved α1α1α2 NC1 trimer (PDB code 1LI1) (21) as the search model. One scNC1 polypeptide was found per asymmetric unit (*V_M_* = 2.6 Å^3^/Da; solvent content = 53% (40)). Refinement of the sc121 NC1-trimer was carried out using Phenix (41) with TLS restraints. The models were manually adjusted between each refinement cycle using Coot (42). Model geometry assessed using MolProbity (43) showed 97.5% of the residues in the favored region and 2.5% in the additionally allowed region, with none in the outlier regions. The final data collection and refinement statistics are shown in Table S1. Model superimpositions were done using LSQ Superpose function in Coot (42).

### Oligomeric state analysis

Size-exclusion chromatography of tissue-extracted NC1 hexamer and the recombinant sc121-NC1 hexamer was conducted with a Superdex 200 Increase 10/300GL gel-filtration column (GE Healthcare), using an ÄKTA purifier (GE Healthcare) at a 0.5 ml/min flow rate. Two eluants were used: 25 mm Tris-HCl, pH 7.5, with 150 mm NaCl (TBS, Cl^−^-reach buffer), and 25 mm Tris acetate, pH 7.5, with 150 mm sodium acetate (TNA, Cl^−^-depleted buffer). Eluting proteins were monitored by *A*_280_. Apparent sizes were calculated using a calibration curve where logarithm of the molecular mass was plotted against normalized retention volume (44) of protein standards (Bio-Rad). The area under hexamer peak was integrated using Unicorn software (GE Healthcare) and expressed as a percentage of the total peak area for quantitation of hexamer assembly.

Sedimentation equilibrium measurements were performed with a Beckman model XLA analytical ultracentrifuge. Samples of 0.3 mg/ml concentration were in TBS or TNA buffers. Runs were carried out at 4 °C in an An60-Ti rotor using 12-mm cells and Epon two-channel centerpieces. The rotor speeds used were 9,000 and 10,000 rpm for TBS and TNA buffers, respectively, and equilibria were reached after 48 h. The SEDNTERP (45) program was used to calculate *v*_bar_ = 0.725 cm^3^ g^−1^, and densities of TBS (1.0051 g cm^−3^) and TNA (1.0054 g cm^−3^) buffers. Data analysis was performed using the SEDFIT and SEDPHAT software (46, 47).

The sample preparation for atomic force microscopy was done on mica (Highest Grade V1 AFM Mica Discs, 10 mm, Ted Pella). The samples in TBS or TNA buffers were diluted into a ∼1–2 μg/ml solution, and 50 μl was deposited onto freshly cleaved mica. After a 30-s incubation period, the excess unbound proteins were washed with ultrapure water for ∼10 s, and the mica was dried immediately under filtered air. All proteins were imaged under dry conditions, and the solution conditions of the samples refer to the conditions in which they were deposited onto mica. AFM imaging was done with an Asylum Research MFP-3D atomic force microscope using AC tapping mode in air. AFM tips with a 160 kHz resonance frequency and 5 newtons/m force constant (MikroMasch, HQ: NSC14/AL BS) were used.

### Secondary structure comparison and thermal stability

Far-UV CD spectra were recorded on a Jasco model J-810 spectrometer equipped with Peltier temperature control unit (JASCO Corp.) using a quartz cell of 1-mm path length at 20 °C. The spectra were normalized for concentration and path length to obtain the mean molar residue ellipticity. Thermal scanning curves were recorded at 230 nm with the heating rate of 0.5 °C/min.

### Hexamer assembly analysis

*In vitro* assembly of the sc121 NC1-trimers and tissue-extracted monomers at 1 mg/ml concentration in 25 mm Tris acetate buffer, pH 7.5, was induced by adding NaCl at 150 mm final concentration and incubated for 24 h at 37 °C unless specified otherwise. The products of assembly were fractionated and analyzed on size-exclusion chromatography.

### Data presentation, fitting, and analysis

3D images were generated using Blender (www.blender.org).^4^ Protein structure figures were generated using PyMOL (9). Experimental data fitting was done using the Gnuplot program (www.gnuplot.info).^4^ Plots were visualized with the Grace program (http://plasma-gate.weizmann.ac.il/Grace/).^4^ Protein structure figures were generated using PyMOL (9). Figure assembly and labeling were done using GIMP and Inkscape software.

## Author contributions

V. P. and S. P. B. formal analysis; V. P., R. B., E. N. P., A. A.-S., N. R. F., and S. P. B. investigation; V. P. and S. P. B. methodology; V. P., R. B., E. N. P., N. R. F., A. L. F., B. G. H., and S. P. B. writing-review and editing; A. L. F., B. G. H., and S. P. B. conceptualization; A. L. F., B. G. H., and S. P. B. writing-original draft; B. G. H. supervision; B. G. H. funding acquisition; B. G. H. project administration; S. P. B. visualization.

## Supplementary Material

Supporting Information

## Footnotes

4 Please note that the JBC is not responsible for the long-term archiving and maintenance of this site or any other third party hosted site.

## Footnotes

3 The abbreviations used are:

BM: basement membrane
ECM: extracellular matrix
PDB: Protein Data Bank
SEC: size-exclusion chromatography
RMSD: root mean square deviation
AFM: atomic force microscopy.
