# Supplementary material for: A chloride ring is an ancient evolutionary innovation mediating the assembly of the collagen IV scaffold of basement membranes
Source: J Biol Chem. 2019 Mar 28;294(20):7968–81. doi: 10.1074/jbc.RA119.007426 (PMC6527180; doi:10.1074/jbc.RA119.007426)
Supplement: Supporting Information [file supp_294_20_7968__index.html]

A chloride ring is an ancient evolutionary innovation mediating the assembly of the collagen IV scaffold of basement membranes — A chloride ring mediates assembly of collagen IV scaffold — A chloride ring is an ancient evolutionary innovation mediating the assembly of the collagen IV scaffold of basement membranes — A chloride ring mediates assembly of collagen IV scaffold — Supporting Information 

# A chloride ring is an ancient evolutionary innovation mediating the assembly of the collagen IV scaffold of basement membranes

## Supporting Information

- Supporting Information (to be published online) - Supplemental Figures and Tables
