# Supplementary material for: A chloride ring is an ancient evolutionary innovation mediating the assembly of the collagen IV scaffold of basement membranes
Source: J Biol Chem. 2019 Mar 28;294(20):7968–81. doi: 10.1074/jbc.RA119.007426 (PMC6527180; doi:10.1074/jbc.RA119.007426)
Supplement: Supporting Information [file supp_RA119.007426_142964_1_supp_306423_pjzqhs.docx]

**Supplemental Figures and Tables:**

**Figure S1.** **Single chain strategy to produce a sc121 NC1-trimer.** (A) In all collagen IV NC1 domains (α1-α6), two C4 subdomains are connected by short linkers (underlined). Similar native-like connecting sequences were designed to connect α1 and α2 NC1 domains (B) and α2 and α1 NC1 domains (C) in a single construct. Several flanking residues (stroked through) were excluded from the final sequence to match the length of native linkers. Artificially introduced linkers GTG and APG between chains are shown in red. Identical residues in native and native-like C4-C4 connections are marked with asterisk.

**Figure S2**. **Amino acid sequence of the single-chain α121 NC1 trimer (sc121 NC1-trimer) construct.** Numbering corresponds to the crystal structure. Native APA linkers connecting C4 subdomains within each chain are shown in underlined bold. Artificially introduced linkers GTG and APG between chains are shown in bold red and underlined.


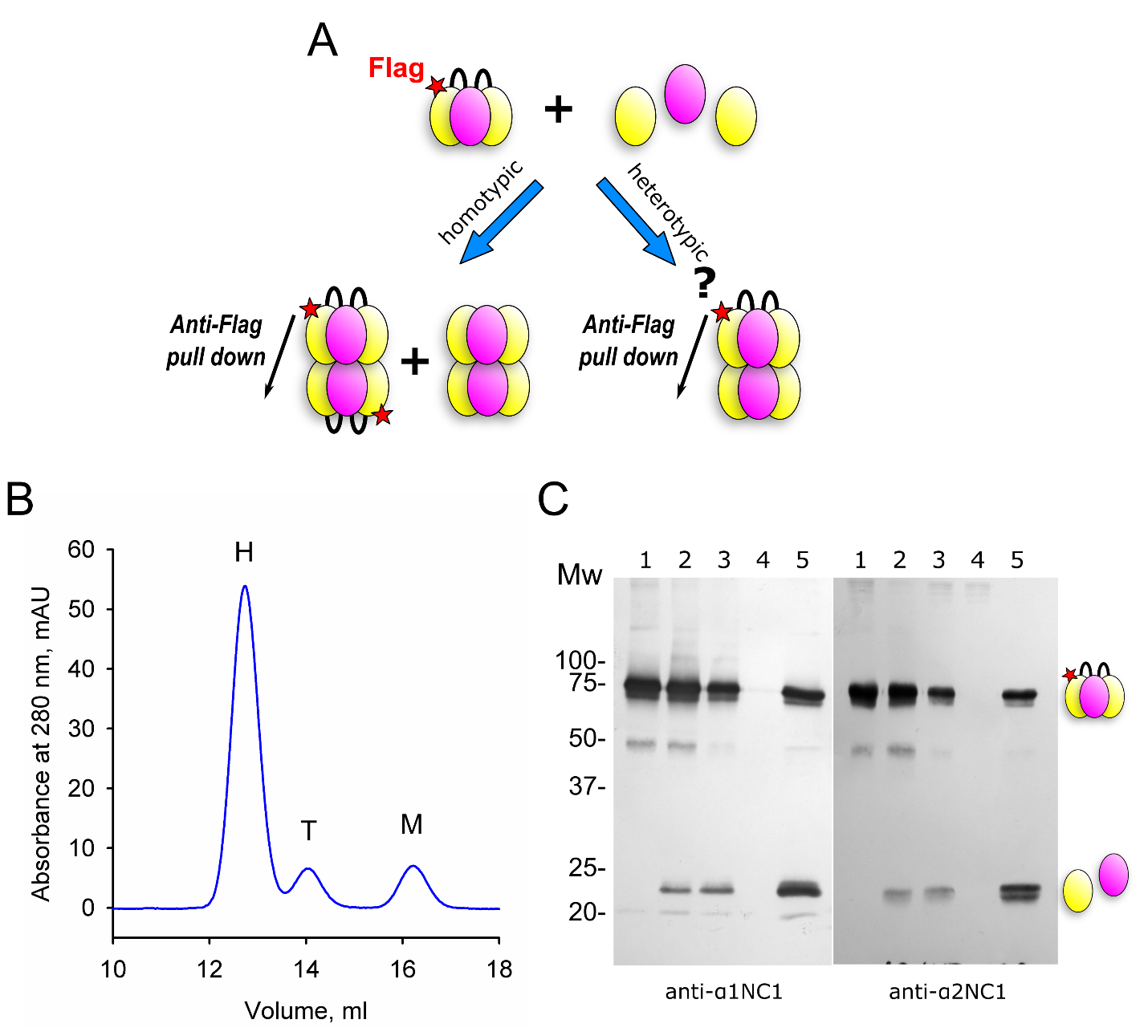


**Figure S3.** **Co-assembly of sc121 NC1-trimer and monomeric NC1.** (A) Two possible ways of re-assembly of recombinant sc121 NC1-trimer with native α1 and α2 NC1 monomers from LBM. (B) After Cl^-^-induced re-assembly, the samples were run over the size-exclusion column to isolate hexamers (H) from trimers (T) and monomers (M) for compositional analysis. (C) Western-blot analysis using anti-α1 (left panel) and anti-α2 (right panel) NC1 antibodies. Samples for lanes 1-4 were hexamers immunoprecipitated using anti-Flag beads to pull down Flag-tagged sc121 NC1-trimer. Lane 1 – mixture of pre-formed sc121-NC1 and LBM hexamers; lane 2 – 1:1 weight mixture of sc121 NC1-trimer and LBM monomers; lane 3 – 1:3 weight mixture of sc121 NC1-trimer and LBM monomers; lane 4 – anti-Flag beads only; lane 5 – sc121 NC1-trimer and LBM hexamer standards. Flag pull-down shows formation of the heterotypic NC1 hexamers.


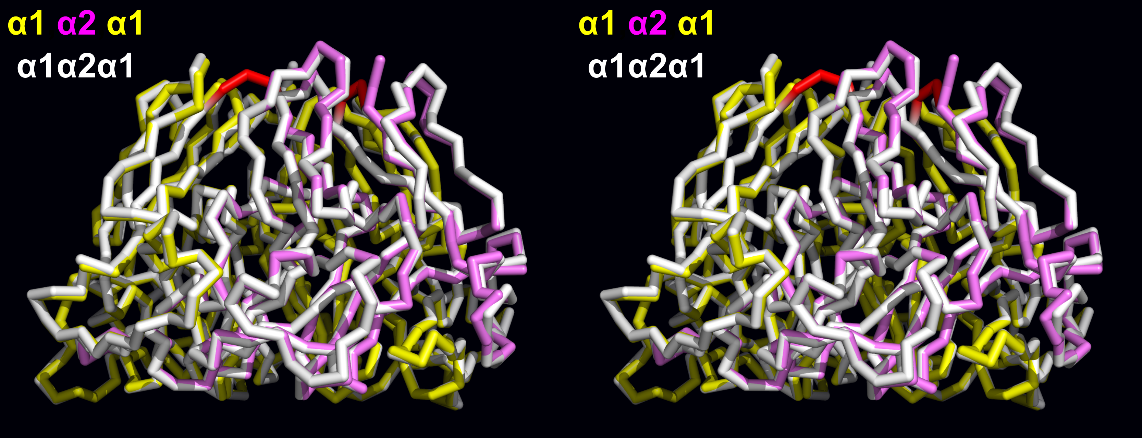


**Figure S4.** **Crystal structure** **comparison of recombinant single chain NC1 trimer vs tissue derived NC1 domains.** Superposition of sc121 NC1-trimer (white) with previously reported structure of the tissue-derived NC1 trimer (PDB 1t61, α1 chains are in yellow, α2 chain in violet) shown as a stereo-pair with wireframe backbone. Artificial linkers are shown in red.


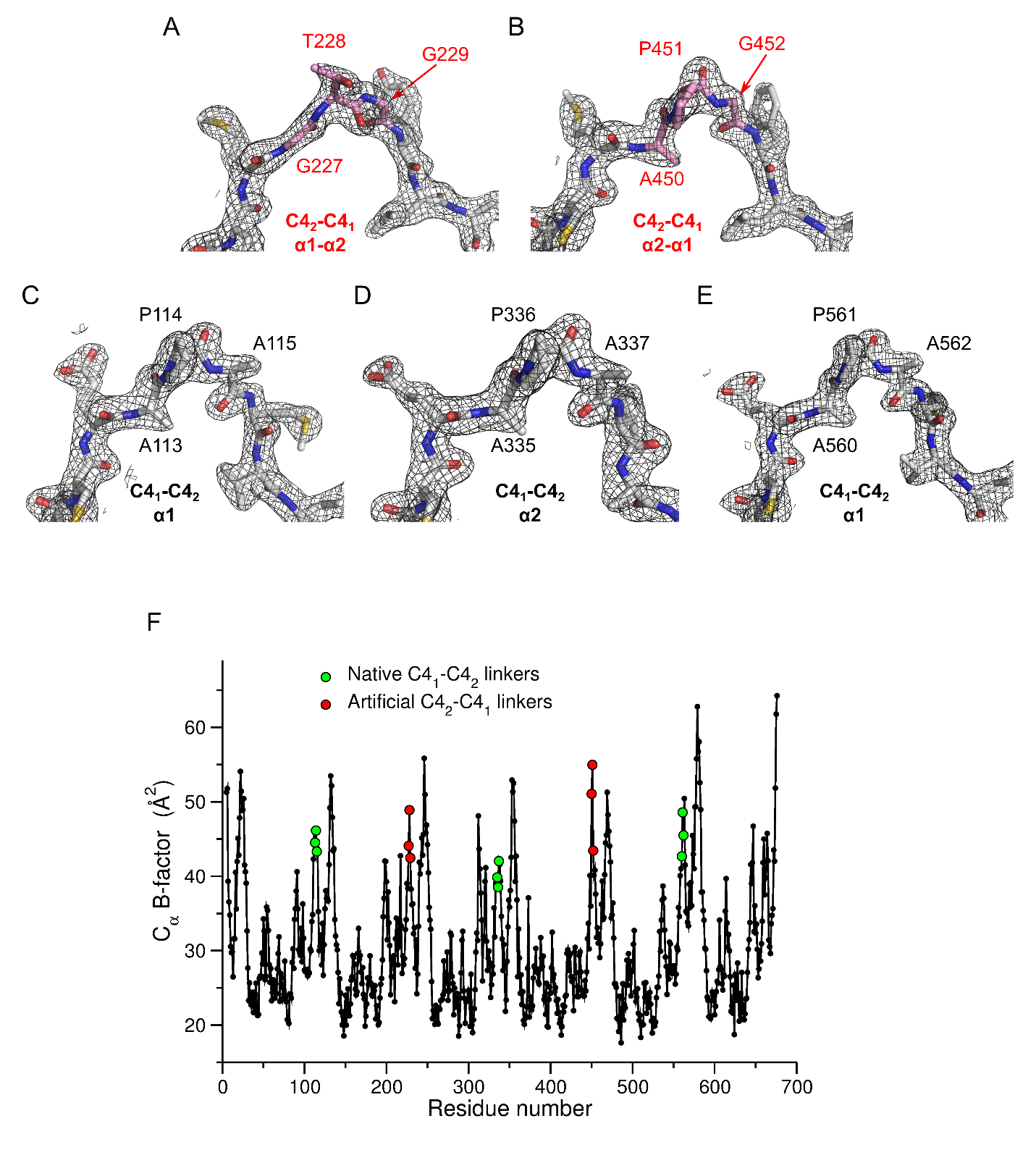


**Figure S5.** **Electron density maps of artificial and native linkers.** (A) and (B) artificial linkers GTG and APG (carbon atoms are shown in pink). (C), (D) and (E) native C4_1_-C4_2_ linkers. The 2F_o_-F_c_ map at σ=1 is represented by the black meshwork. **Figure S6.** **Mean square displacement (B factor) of Cα atoms.** B factor values for native and artificial linkers are comparable as depicted by green and red circles, respectively.


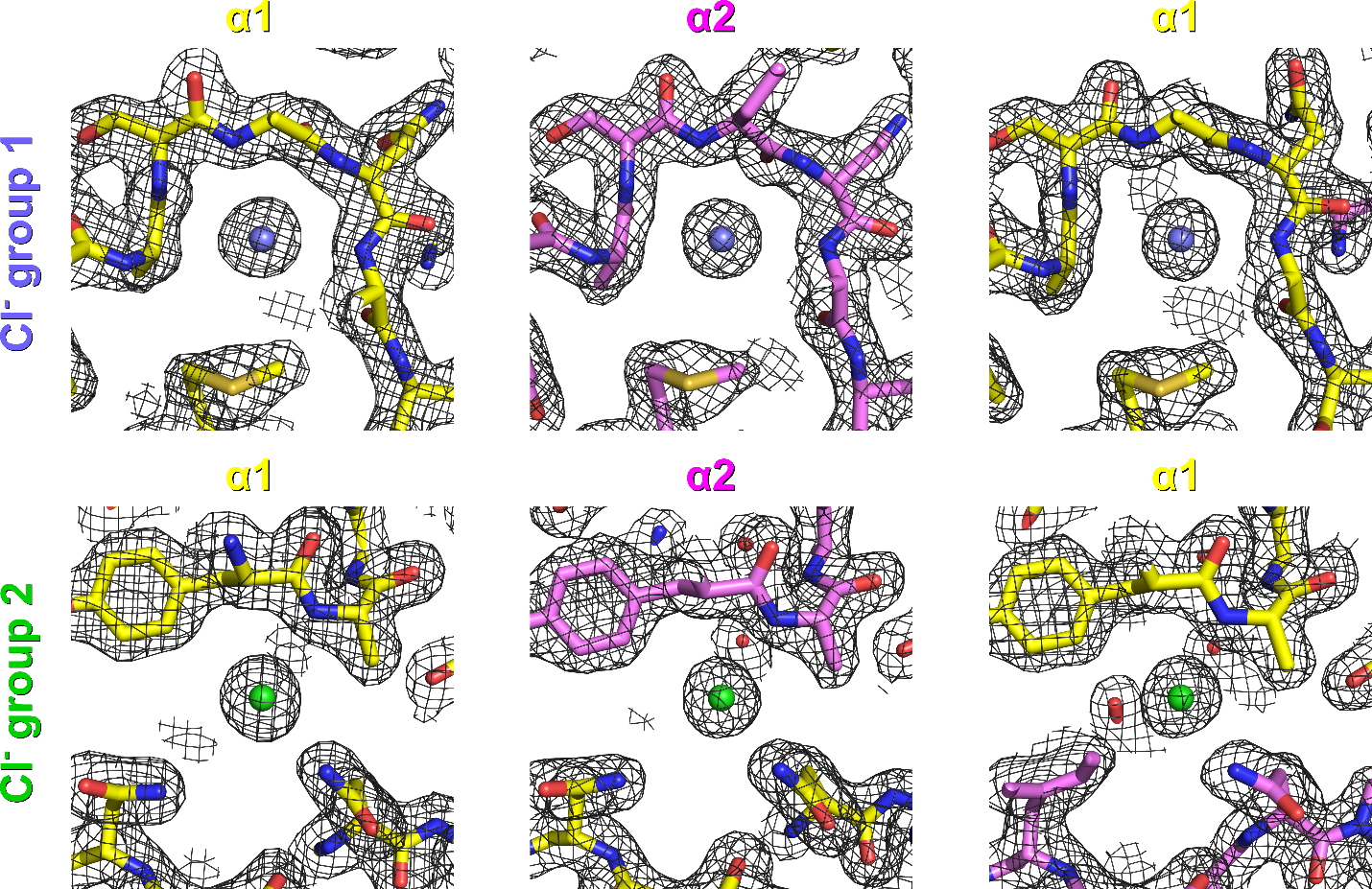


**Figure S6.** **Electron density maps of chloride ions.** Cl^-^ group 1 (blue spheres) and group 2 (green spheres) are well coordinated and have comparable densities. The black meshwork represents the 2F_o_-F_c_ electron density map at σ=1. Carbon atoms of α1 chains are shown in yellow, and those of α2 are in violet.


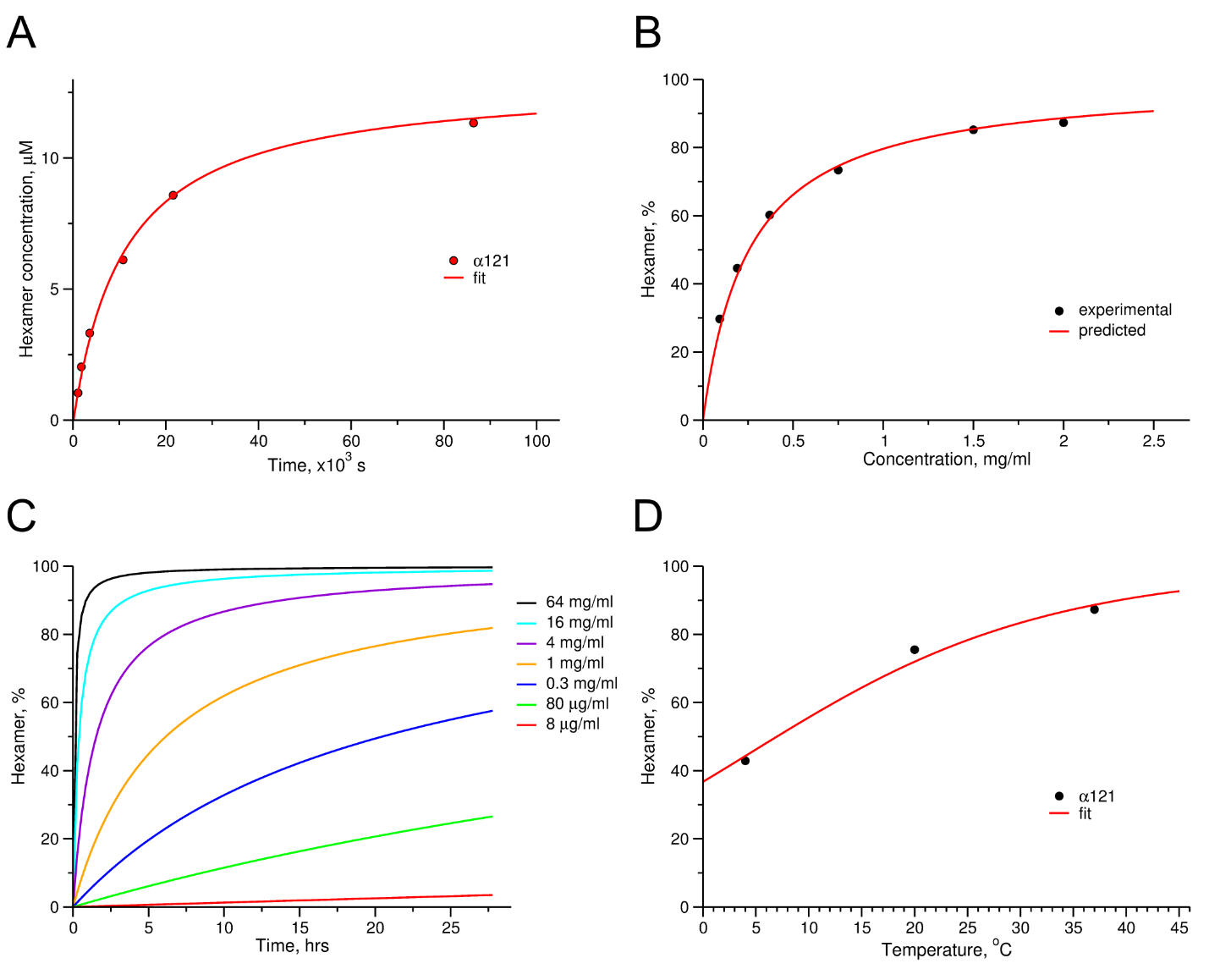


**Figure S7**. **Hexamer assembly from the sc121 NC1-trimer is described by the bimolecular assembly model and predicts concentration-dependent data.** (A) Experimental data were fit using the second-order reaction equation 3. (B) The determined rate constant of *k*_a_=3.45 ± 0.12 M^-1^ s^-1^ was used to predict concentration-dependent formation of the hexamer, in excellent agreement with the experimental data. (C) This model can be used to predict hexamer formation kinetics for a wide range of NC1 concentrations. (D). Temperature-dependent data was fitted using the model equation and a single variable parameter E_a_, activation energy.

**Table S1. Data collection and refinement statistics**

| Data collection statistics |  |
| --- | --- |
| Wavelength (Å) | 0.9786 |
| Resolution range (Å)^a^ | 39.90-1.90 (1.93-1.90) |
| Space group | P4_1_ 2_1_ 2 |
| Unit cell dimension (Å) | |
| a, b | 121.2 |
| c | 106.1 |
| Unit cell angle (°) | |
| α, β, γ | 90 |
| Total reflections | 558,146 |
| Redundancy | 8.9 (9.0) |
| Completeness (%)^a^ | 99.9 (100) |
| R_meas_ (%)^a, b^ | 12.9 (47.0) |
| I/σI^b^ | 9.5 (3.7) |
|  |  |
| Refinement statistics |  |
| Unique reflections | 62,515 |
| R_work_ (%) | 14.8 (21.1) |
| R_free_ (%)^a, b^ | 17.5 (25.7) |
| Coordinates ESU (Å) | 0.18 |
| Root mean square deviations | |
| Bond distance (Å) | 0.01 |
| Bond angles (°) | 1.66 |
| Chiral centers (Å^3^) | 0.05 |
| Planar groups (Å) | 0.01 |
| Number of atoms | 5,771 |
| Protein atoms | 5,225 |
| Ligand/ion atoms | 106 |
| Water molecules | 440 |
| Average B-factor (Å^2^) | 35.2 |
| Protein B-factor (Å^2^) | 34.4 |
| Ligand/ion B-factor (Å^2^) | 50.8 |
| Water B-factor (Å^2^) | 41.4 |
| Ramachandran statistics | |
| Favored (%) | 97.5 |
| Additionally allowed (%) | 2.5 |
| Outliers (%) | 0 |

^a^ Data for highest resolution shell are given in parentheses

^b^ R_meas_ =$\sum_{h} \sum_{i} \sqrt{\frac{N_{h}}{N_{h}-1}}\left| I_{hi}-\left\langle I_{h} \right\rangle\right|/\sum_{h} \sum_{i} \left\langle I_{h} \right\rangle$^c^ 5% of data excluded from refinement

**Table S2.** **Chloride ions coordination geometry.** Shown are distances between Cl^-^ ions and coordinating atoms. Atoms that belong to or are coordinated by the opposite trimer are marked by asterisks, while atoms coordinated by both trimers are marked by #.

Group 1

| Cl^-^ #1 | | Cl^-^ #2 | | Cl^-^ #3 | |
| --- | --- | --- | --- | --- | --- |
| A74, Cα | 3.6 Å | A298, Cα | 3.6 Å | A521, Cα | 3.8 Å |
| R76, N | 3.5 Å | R300, N | 3.3 Å | R523, N | 3.5 Å |
| D78, N | 3.3 Å | D302, N | 3.4 Å | D525, N | 3.2 Å |
| *R179, Nη1 | 4.7 Å | *R626, Nη1 | 3.8 Å | *R402, Nη1 | 3.1 Å |
| H_2_O, O | 3.2 Å | H_2_O, O | 3.1 Å | H_2_O, O | 3.2 Å |
| ^#^H_2_O, O | 3.1 Å | ^#^H_2_O, O | 3.1 Å | ^#^H_2_O, O | 3.4 Å |

Group 2

| Cl^-^ #4 | | Cl^-^ #5 | | Cl^-^ #6 | |
| --- | --- | --- | --- | --- | --- |
| F64, Cɛ1 | 3.9 Å | F511, Cɛ1 | 3.9 Å | Y288, Oη | 3.0 Å |
|  |  |  |  | Y296, Cε2 | 3.8 Å |
| N66, Nδ2 | 3.4 Å | N513, Nδ2 | 3.2 Å | N290, Nδ2 | 3.3 Å |
| R76, Nη1 | 5.4 Å | R523, Nη1 | 5.1 Å | R300, Nη1 | 4.4 Å |
| Y636, Oη | 3.4 Å | Y412, Oη | 3.1 Å | Y189, Oη | 3.1 Å |
| *Y185, Cδ1 | 3.9 Å | *Y408, Cδ1 | 3.7 Å | *Y408, Cδ1 | 3.7 Å |
| *Y185, Cβ | 4.1 Å | *Y408, Cβ | 3.8 Å | *Y408, Cβ | 3.8 Å |
| *A186, N | 3.3 Å | *A409, N | 3.1 Å | *A409, N | 3.1 Å |
| *A186, Cβ | 3.8 Å | *A409, Cβ | 3.8 Å | *A409, Cβ | 3.8 Å |
| H_2_O, O | 3.5 Å | H_2_O, O | 3.4 Å |  |  |
| *H_2_O, O | 3.3 Å | *H_2_O, O | 3.2 Å |  |  |
